# Temporal trends of the association between ambient temperature and hospitalisations for cardiovascular diseases in Queensland, Australia from 1995 to 2016: A time-stratified case-crossover study

**DOI:** 10.1371/journal.pmed.1003176

**Published:** 2020-07-21

**Authors:** Peng Lu, Guoxin Xia, Qi Zhao, Rongbin Xu, Shanshan Li, Yuming Guo

**Affiliations:** 1 School of Public Health and Management, Binzhou Medical University, Yantai, Shandong, China; 2 Department of Epidemiology and Preventive Medicine, School of Public Health and Preventive Medicine, Monash University, Melbourne, Victoria, Australia; 3 School of Medicine, Binzhou Medical University, Yantai, Shandong, China; Seoul National University, REPUBLIC OF KOREA

## Abstract

**Background:**

In the context of global warming, studies have turned to assess the temporal trend of the association between temperature and health outcomes, which can be used to reflect whether human beings have adapted to the local temperature. However, most studies have only focused on hot temperature and mortality. We aim to investigate the temporal variations in the association between ambient temperature and hospitalisations for cardiovascular diseases in Queensland, Australia from 1995 to 2016.

**Methods and findings:**

We obtained data on 1,855,717 cardiovascular hospitalisations (mean age: 65.9 years, 42.7% female) from all 443 postal areas in Queensland, Australia between January 1, 1995 and December 31, 2016. Grid-level meteorological data were downloaded from scientific information for landowners. We used a time-stratified case-crossover design fitted with a conditional quasi-Poisson regression model and time-varying distributed lag nonlinear model (DLNM) to evaluate the association between temperature and cardiovascular hospitalisations and the temporal trends of the associations. Stratified analyses were performed in different age, sex, and climate zones. In all groups, relative risks (RRs) of cardiovascular hospitalisations associated with high temperatures (heat effects) increased, but cold effects showed a decreasing trend from 1995 to 2016. The increasing magnitude of heat effects was larger (*p* = 0.002) in men than in women and larger (*p* < 0.001) in people aged ≤69 years than in those aged ≥70 years. There was no apparent difference amongst different climate zones. The study was limited by the switch from ICD-9 to ICD-10 coding systems, by being unable to separate first-time hospitalisation from repeated hospitalisations, and possibly by confounding by air pollution or by influenza infections.

**Conclusion:**

The impacts of cold temperatures on cardiovascular hospitalisations have decreased, but the impacts of high temperatures have increased in Queensland, Australia. The findings highlight that Queensland people have adapted to the impacts of cold temperatures, but not high temperatures. The burden of cardiovascular hospitalisations due to high temperatures is likely to increase in the context of global warming.

## Abstract 2

Peng Lu and colleagues investigate associations over time between ambient temperatures and hospitalizations for cardiovascular diseases.

## Author summary

**Why was this study done?:**

- Studies have reported temporal decline in the association between hot temperature and cardiovascular mortality, which indicates that people may have adapted to the warming climate.
- However, little is known about the change in the association between ambient temperature and cardiovascular hospitalisation in recent decades.

**What did the researchers do and find?:**

- Using 1.9 million cardiovascular hospitalisations during 1995–2016 in Queensland, Australia, we developed a model to evaluate the association between temperature and cardiovascular hospitalisations and their trend over time.
- We found that the impact of cold temperature on cardiovascular hospitalisation decreased, but the impact of high temperature on cardiovascular hospitalisations increased from 1995 to 2016. The results were robust amongst different subgroups.
- The increasing heat effect was more serious for men than women and for people ≤69 years than people ≥70 years.

**What do these findings mean?:**

- Given the increased associations between hot temperature and cardiovascular hospitalisation, the acceleration of global warming, and an aging population, we highlight a worrying trend that the burden of cardiovascular healthcare facilities is to increase over time on high-temperature days.
- Heat warning systems should focus more on men and people ≤69 years old, since heat effects on these groups increased more noticeably than for women and people ≥70 years old, respectively.

## Introduction

Cardiovascular diseases remain the leading cause of mortality and morbidity worldwide [1,2]. Extreme temperature is an established risk factor for cardiovascular mortality and morbidity [3]. There is an increasing certainty that the duration, intensity, and spatial extent of extreme temperatures will increase if current emission trends of greenhouse gases continue, which will exacerbate the temperature impacts on cardiovascular system [4]. Recently, studies have turned to assess the temporal trend of the association between temperature and health outcomes, which can be used to reflect whether human beings have adapted to the local temperature. However, most studies have only focused on hot temperature and mortality [5–7]. Only a few studies have investigated the effects of the whole temperature spectrum, and the temporal variations of temperature–mortality associations showed heterogeneous trends [8–12]; amongst these, only one study examined the temperature–cardiovascular mortality relationship [13].

Meanwhile, most studies have only focused on temperature–mortality association. Few studies have investigated the temporal trends of temperature–morbidity (e.g., hospitalisation) associations [14–16]. Far fewer studies investigated the demographic and geographic characteristics of these temporal trends. Zhao and colleagues investigated the temporal variations in the association between heat exposure and hospitalisations in Brazil and found a complicated geographic pattern [14]. Sun and colleagues investigated the temporal variations in temperature–respiratory hospitalisation associations and found an increased susceptibility to heat amongst patients aged <65 years between 2000 and 2016 in Hong Kong [16].

Understanding the temporal change in the temperature–hospitalisation association and the demographic, spatial characteristics can help inform current public health policy and future infrastructure allocation. This is particularly true for Australia, a country where the cardiovascular disease death rates declined before 2010, but the trend has reversed recently [17]. A total of 1.1 million hospitalisations were attributable to cardiovascular diseases in Australia during 2015–2016 [18]. However, there is no study to investigate the temporal variations in the temperature–cardiovascular hospitalisation associations and the influencing factors in Australia.

To fill this research gap, we examined the temporal variations in the association between ambient temperature and cardiovascular hospitalisations in Queensland, Australia, between 1995 and 2016. We also examined effect modifiers as suggested by prior epidemiological evidence [11]: intrinsic factors (age and sex) or extrinsic factors (local climate).

## Methods

This time-stratified case-crossover study is reported following the Strengthening the Reporting of Observational Studies in Epidemiology (STROBE) guidelines (S1 Text). We performed the data analyses for the present study following a prospective analysis plan (S2 Text). Changes in the analysis plan are described in S2 Text.

### Study area

The State of Queensland is located in the northeast of Australia. It is the second largest (1,852,642 square kilometres) and third most populous state, with an estimated population of 4,703,193 [19]. The state has a subtropical to tropical climate with warm, humid summers and mild winters. Queensland includes 443 postal areas (Australian Bureau of Statistics, postal areas Australian Statistical Geography Standard [ASGS] Edition 2016). The base map of Queensland was downloaded from Australian Bureau of Statistics 2016, ASGS.

### Data collection

#### Hospitalisation data

Postcode-level hospitalisation data of Queensland were collected between 1 January 1995 and 31 December 2016 from Queensland Hospital Admitted Patient Data Collection (QHAPDC), Queensland Health. The QHAPDC collects demographic data and clinical information on all patients admitted to public and licensed private hospitals and private day surgeries in Queensland, Australia. Information included date of admission, sex, age, postcode, and primary diagnosis of all cardiovascular diseases. The diagnosis of cardiovascular diseases follows the International Classification of Diseases, 9^th^ Revision (ICD-9) codes: 390–459 (for the period January 1, 1995–June 30, 1999) or 10^th^ Revision (ICD-10) codes: I00–I99 (for the period July 1, 1999–December 31, 2016). We also separated the hospitalisation data according to different sex (male and female) and age (0–59, 60–69, 70–79, ≥80 years). Ethics approval was not required for our analysis of the anonymised data from QHAPDC.

#### Meteorological data

Data on daily maximum and minimum temperatures were downloaded from scientific information for landowners (SILO), hosted by the Science and Technology Division of Queensland Government’s Department of Environment and Science (www.des.qld.gov.au/). The climatic data sets are 0.05° × 0.05° (about 5 km × 5 km) grid data constructed from observational weather data from the Australian Bureau of Meteorology. Daily mean temperature, approximated as the mean of daily maximum and minimum temperatures, was used to analyse the temperature–hospitalisation associations [14]. Daily relative humidity data during the study period were also downloaded from SILO. Daily climatic data were linked to hospitalisations according to date and postcode area (the average value of all grids covering the postcode area). According to Census of Population and Housing (Australian Bureau of Statistics, Australia, 2016), there were 443 postal areas in Queensland, Australia. We calculated the mean temperature of each postcode area during the study period. Then, we trisected the postal areas according to their mean temperatures from high to low, i.e., 148 hot climate postcode areas (daily temperature ranges: −4.0 °C to approximately 48.4 °C), 147 moderate climate postcode areas (daily temperature ranges: −4.5 °C to approximately 48.0 °C), and 148 cold climate postcode areas (daily temperature ranges: −8.9 °C to approximately 46.8 °C) (S1 Fig).

### Statistical analysis

#### Temperature–hospitalisation association

We used a time-stratified case-crossover design to control for the long-term trend and seasonal trend. We performed conditional quasi-Poisson regression with distributed lag nonlinear model (DLNM) to estimate the temperature–hospitalisation associations, which were reported as relative risks (RRs). We used the lag day up to 21 days to fully capture the delayed effects and harvesting effects [13]. Based on previous studies, we fitted the exposure–response association using a natural cubic spline with 3 degrees of freedom (*df*) and fitted the lag–response association using a natural cubic spline with 4 *df* [20]. The impacts of relative humidity were controlled for using a natural cubic spline with 3 *df* [20]. The quasi-Poisson regression model was as follows:

Log(E(Y))=α+β*stratum+cb(temp)+ns(rh)+holiday,

where *Y* is the daily counts of hospitalisation and *α* is the intercept. Stratum was defined as the same days of the week in the same month of the same year and same postcode; *cb* is the cross-basis function to fit the nonlinear and lagged effect of daily mean temperature (*temp*); *ns* is the natural cubic spline function of daily mean relative humidity (*rh*); and *holiday* is a binary variable to control for public holidays.

#### Temporal trends in the temperature–hospitalisation association

We examined the temporal variation of associations between ambient temperature and hospitalisations for cardiovascular diseases with the time-varying DLNM [21]. We extended the above time-constant model to the time-varying DLNM by including a linear interaction between time (a sequence from the first day to the last day of study period) and *cb*(*temp*). We compared the RRs in the middle of year 1995 and the middle of year 2016 to examine whether there was a difference between the 2 years.

To calculate minimum hospitalisation temperature (MHT), we first get the percentile scale of temperature corresponding to the lowest risk of daily hospitalisation in the temperature–hospitalisation association curve in Queensland, as applied by previous studies [22,23]. The MHT of each postcode area was calculated according to the percentile temperature corresponding to the minimum hospitalisations in the whole of Queensland. The MHT of Queensland was defined as the average MHT of all postcode areas. The overall MHT was estimated from the model with no interaction. Cold effect was defined as the RR at first percentile of temperature against the tenth percentile of temperature, and heat effect was defined as the RR at the 99^th^ percentile of temperature against the 90^th^ percentile of temperature.

We used the Wald test to check the significance of the temporal change. Meta-regression was performed to examine whether there was a significant difference of the temporal change amongst subgroups.

### Sensitivity analyses

A series of sensitivity analyses were conducted to examine the robustness of our findings. We varied the number of lag days from 0–21 to 0–28 days for temperature to check whether using 21 lag days was sufficient to examine delayed effects and harvesting effects. We changed the *df* for meteorological variables from 2 to 4. We tested the lag–response curves at the first percentile of temperature against the tenth percentile of temperature and the 99^th^ percentile of temperature against the 90^th^ percentile of temperature for 1995 and 2016. We also analysed the association between temperature and hospitalisation for cardiovascular diseases in every 5 years with 3 years overlapping to check whether our time-varying DLNM was robust or not. To be consistent with previous studies, we analysed the RRs of the associations between the 2.5^th^, 5^th^, 95^th^, and 97.5^th^ percentile of temperature and the hospitalisation amongst different subgroups. We also checked the association between different temperature metrics (maximum, minimum, and apparent temperature) and hospitalisation. We added log-transformed population as an offset to check if there was any change in results.

R software (version 3.5.1) was used for all data analyses. The ‘gnm’ and ‘dlnm’ packages were used to fit conditional a quasi-Poisson regression model and the DLNM, respectively.

## Results

**Fig 1:**
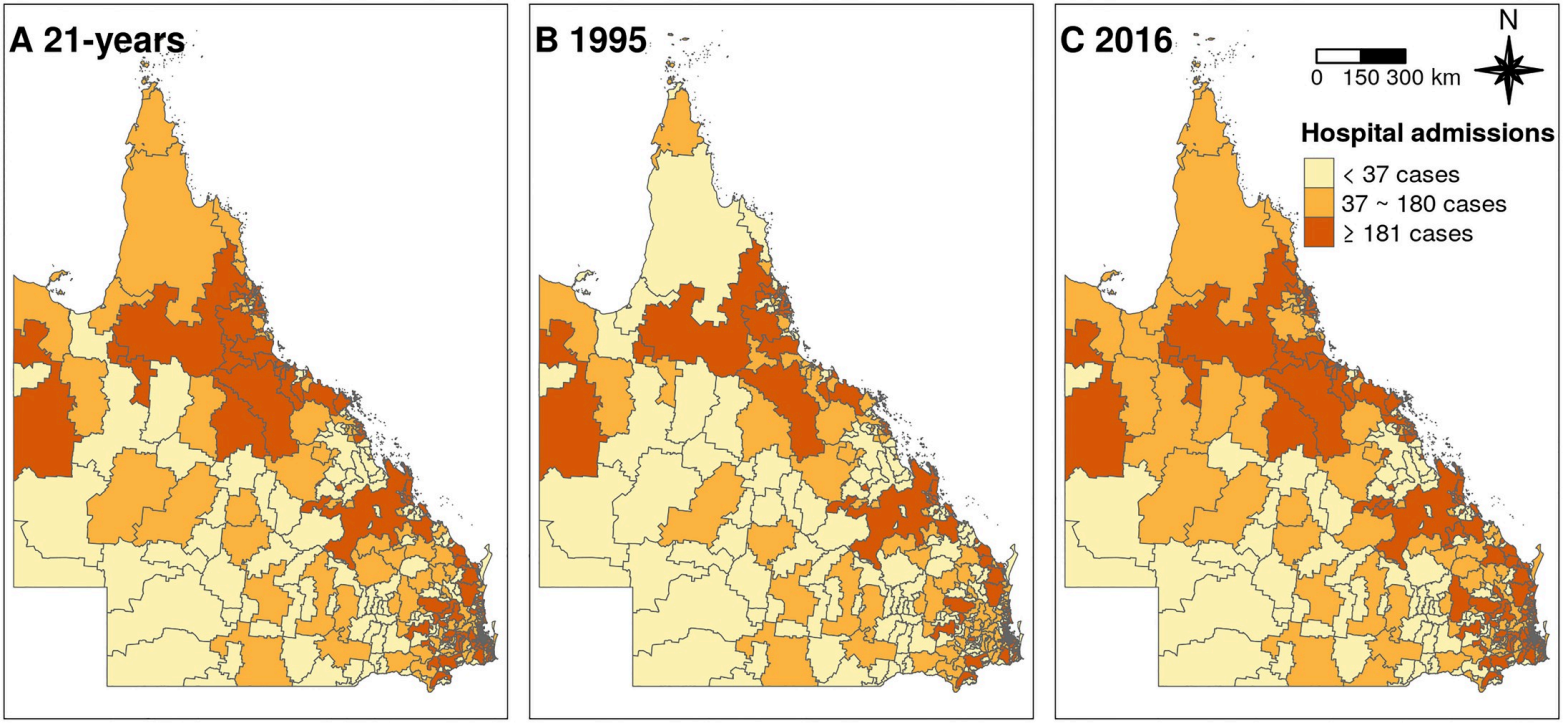
(A) Distribution of annually average hospitalisations for cardiovascular diseases in Queensland, Australia between 1995 and 2016. (B) Distribution of average hospitalisations for cardiovascular diseases in Queensland, Australia in 1995. (C) Distribution of average hospitalisations for cardiovascular diseases in Queensland, Australia in 2016. The base map was downloaded from ASGS, Australian Bureau of Statistics (https://www.abs.gov.au/AUSSTATS/abs@.nsf/DetailsPage/1270.0.55.003July%202016?OpenDocument). The base map was open access. ASGS, Australian Statistical Geography Standard.

Table 1 shows the descriptive results. The average daily mean temperature of the 443 postal areas was 21.0°C (range: −8.9 °C to 48.4 °C) between 1995 and 2016 in Queensland. The daily mean temperature has increased from 20.9 °C in 1995 to 21.7 °C in 2016. In addition, the minimum, 25^th^, 50^th^, 75^th^, and maximum temperatures all increased from 1995 to 2016. During the study period, there were 1,855,717 cardiovascular hospitalisations. The distribution of annual average hospitalisations for cardiovascular diseases over the 21-year study period is shown in Fig 1A. Fig 1B and 1C show the distribution of hospitalisations for cardiovascular diseases in 1995 and in 2016, respectively. In comparison with 1995, the hospital admissions for cardiovascular diseases increased dramatically in 2016.

**Table 1:** Distribution of enrolled hospitalisations and temperature features in the 443 postal areas between 1995 and 2016 in Queensland, Australia.

	Subgroup	Number of Cases	Average Postal Area Temperatures (°C)					
			Mean	Minimum	25^th^	50^th^	75^th^	Maximum
Total		1,855,717	21.0	−8.9	17.5	21.6	24.6	48.4
	1995	46,730	20.9	−6.7	17.7	21.4	24.4	45.2
	2016	123,477	21.7	−5.1	18.3	22.4	25.3	45.7
Climate								
	Hot	548,047	23.1	−4.0	19.8	23.8	26.6	48.4
	Mild	861,612	20.7	−4.5	17.3	21.1	24.0	48.0
	Cold	446,058	19.3	−8.9	15.3	19.8	23.2	46.8
Sex								
	Men	1,063,325	–	–	–	–	–	–
	Women	792,392	–	–	–	–	–	–
Age (years)								
	0–59	579,046	–	–	–	–	–	–
	60–69	403,951	–	–	–	–	–	–
	79–80	459,754	–	–	–	–	–	–
	80+	412,966	–	–	–	–	–	–

Note: climate regions were divided according to the daily mean temperature of postal areas.

**Fig 2:**
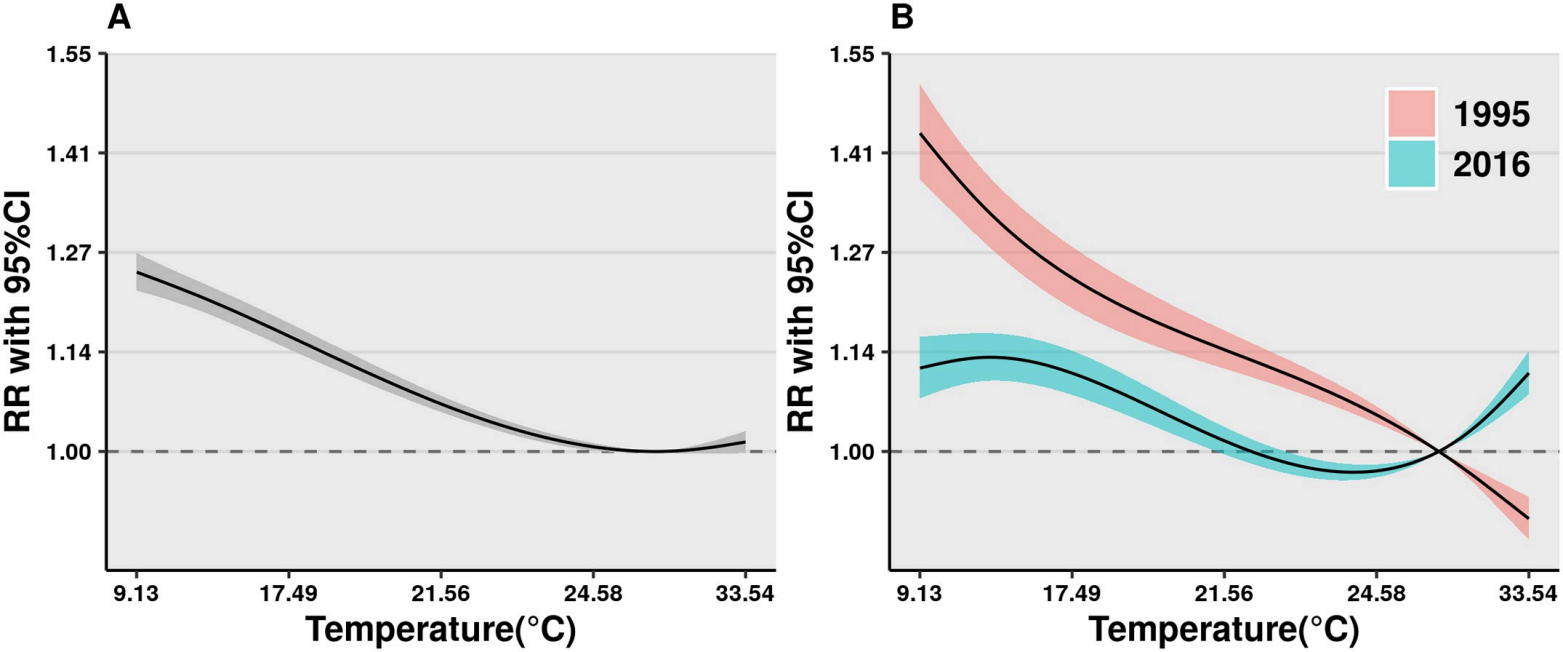
(A) The average cumulative exposure–response relationships between temperature and hospitalisations for cardiovascular diseases along lag 0–21 days between 1995 and 2016 and (B) the cumulative exposure–response relationships between temperature and hospitalisations for cardiovascular diseases along lag 0–21 days in 1995 (blue) and 2016 (pink). Note: polygon areas represent 95% CI. CI, confidence interval; RR, relative risk.

Fig 2A shows the temperature–cardiovascular hospitalisation relationships (lag 0–21 days) predicted using the time-constant DLNM, with 95% confidence interval (95% CI). The average relationship was nonlinear in Queensland, with MHT located at 25.9 °C. Cold effects (first percentile of temperature against tenth percentile of temperature) were significantly associated with the increased risks of hospitalisations for cardiovascular diseases (RR 1.02 [95% CI: 1.01, 1.03], *p* < 0.001). Heat effects (99^th^ percentile of temperature against 90^th^ percentile of temperature) were insignificantly associated with increased risks of hospitalisations for cardiovascular diseases (RR 1.01 [95% CI: 1.00, 1.02]).

Fig 2B shows the temperature–cardiovascular hospitalisation relationships in 1995 (pink) and in 2016 (blue), with 95% CI, modelled using the time-varying DLNM. There were strong (*p* < 0.001, Wald test) differences in the cold and heat effects in 1995 and in 2016. In 1995, the association between temperature and hospitalisations for cardiovascular diseases showed a nearly linear relationship, with RRs decreased with temperature increasing. This indicated there was no heat effect. Cold effect was positively associated with hospitalisations for cardiovascular diseases (RR 1.06 [95% CI: 1.04, 1.09], *p* < 0.001). In 2016, the association was U-shaped. Heat effects in 2016 were significantly associated with the increased risk of hospitalisations for cardiovascular diseases with RR 1.07 (95% CI: 1.05, 1.09), *p* < 0.001.

Table 2 shows the MHTs for different subgroups. The MHT in 1995 was the highest temperature. The MHTs of all subgroups in 2016 was lower than that of the overall MHTs.

**Table 2:** MHT (°C) for cardiovascular diseases amongst different subgroups between 1995 and 2016.

	Subgroups	Overall	1995	2016
	Total	25.9	33.5	24.3
Sex	Female	26.1	33.5	24.5
	Male	25.6	33.5	24.0
Age (years)	0–59	24.9	33.5	24.0
	60–69	26.1	33.5	24.0
	70–79	25.9	33.5	24.7
	80+	33.5	33.5	23.6
Climate	Cold	25.4	33.5	24.0
	Mild	25.4	33.5	24.3
	Hot	33.5	33.5	24.0

**Abbreviations**: MHT, minimum hospitalisation temperature.

Table 3 shows the RRs of the associations between cold temperatures and the hospitalisations for cardiovascular diseases amongst different subgroups. The magnitude of cold effects decreased significantly in all subgroups except in the ≥80 years age group from 1995 to 2016.

**Table 3:** RRs (95% CIs) of the associations between cold temperatures and hospitalisation for cardiovascular diseases amongst different subgroups.

		Cold (First Percentile of Temperature against Tenth Percentile) Effects			*p*-Values for Difference between 1995 and 2016
	Subgroups	1995–2016	1995	2016	
Total	Total	1.02 (1.01, 1.03)	1.06 (1.04, 1.09)	0.99 (0.97, 1.01)	<0.001
Age (years)	0–59	1.01 (1.00, 1.03)	1.06 (1.03, 1.09)	0.96 (0.94, 0.99)	<0.001
	60–69	0.98 (0.97, 1.00)	1.01 (0.98, 1.04)	0.97 (0.94, 0.99)	0.046
	70–79	1.03 (1.01, 1.04)	1.11 (1.08, 1.14)	0.96 (0.94, 0.99)	<0.001
	80+	1.07 (1.06, 1.09)	1.08 (1.05, 1.12)	1.07 (1.04, 1.09)	0.499
Sex	Female	1.03 (1.02, 1.04)	1.06 (1.04, 1.09)	1.00 (0.98, 1.02)	<0.001
	Male	1.02 (1.01, 1.03)	1.07 (1.04, 1.09)	0.98 (0.96, 1.00)	<0.001
Climate	Cold	1.03 (1.01, 1.05)	1.07 (1.03, 1.11)	1.00 (0.97, 1.03)	0.011
	Mild	1.01 (0.99, 1.03)	1.05 (1.01, 1.08)	0.97 (0.94, 1.00)	<0.001
	Hot	1.04 (1.02, 1.05)	1.09 (1.05, 1.13)	1.00 (0.97, 1.03)	<0.001

**Abbreviations**: CI, confidence interval; RR, relative risk.

Table 4 shows the RRs of the associations between high temperatures and hospitalisations for cardiovascular diseases amongst different subgroups. There was no heat effect in 1995. The heat effects increased in all subgroups from 1995 to 2016. The increasing magnitude of heat effect was larger (*p* = 0.002) in men than in women and larger (*p* < 0.001) in the ≤69 years age group than that of other age groups. The comparison of the increasing magnitude of heat effect is shown in S1 Table.

**Table 4:** RRs (95% CIs) of the associations between high temperatures and hospitalisation for cardiovascular diseases amongst different subgroups.

		Heat (99^th^ Percentile of Temperature against 90^th^ Percentile) Effects			*p*-Values for Difference between 1995 and 2016
	Subgroups	1995–2016	1995	2016	
Total	Total	1.01 (1.00, 1.02)	0.94 (0.92, 0.96)	1.07 (1.05, 1.09)	<0.001
Age (years)	0–59	1.02 (1.01, 1.03)	0.94 (0.92, 0.97)	1.09 (1.07, 1.12)	<0.001
	60–69	1.01 (1.00, 1.03)	0.91 (0.88, 0.94)	1.09 (1.06, 1.12)	<0.001
	70–79	1.01 (1.00, 1.02)	0.95 (0.92, 0.97)	1.07 (1.04, 1.10)	<0.001
	80+	1.00 (0.98, 1.01)	0.95 (0.92, 0.99)	1.03 (1.00, 1.05)	<0.001
Sex	Female	1.01 (0.99, 1.02)	0.96 (0.94, 0.99)	1.05 (1.02, 1.07)	<0.001
	Male	1.01 (1.00, 1.02)	0.92 (0.90, 0.95)	1.09 (1.07, 1.11)	<0.001
Climate	Cold	1.01 (1.00, 1.03)	0.94 (0.90, 0.98)	1.08 (1.04, 1.12)	<0.001
	Mild	1.02 (1.01, 1.04)	0.97 (0.94, 1.01)	1.08 (1.04, 1.11)	<0.001
	Hot	0.99 (0.97, 1.00)	0.90 (0.87, 0.93)	1.06 (1.03, 1.09)	<0.001

**Abbreviations**: CI, confidence interval; RR, relative risk.

Sensitivity analyses showed that the association between temperature and hospitalisations for cardiovascular diseases in every 5 years with 3 years overlapping showed similar association patterns as the pattern predicted from the time-varying DLNM model (S2 Fig). Using 22–28 lag days for temperature (S3 Fig) or 3–5 *df* for meteorological variables (S4 and S5 Figs) did not substantially change the effect estimates of the associations between temperature and hospitalisations for cardiovascular diseases. The lag–response relationships between temperature (cold/heat) and hospitalisations predicted for 1995 and 2016 is shown in S6 and S7 Figs. The temperature–hospitalisation association became stable at 21 lag days. The RRs of the associations between the 2.5^th^, 5^th^, 95^th^, and 97.5^th^ percentiles of temperature and the hospitalisations amongst different subgroups is shown in S2 and S3 Tables. The maximum, minimum, and apparent temperature–cardiovascular hospitalisation relationships (lag 0–21 days), with 95% CI, is shown in S8–S10 Figs. The results of using different cutoffs of heat and cold exposure and using different temperature metrics are the same as main results. The associations between temperature and cardiovascular hospitalisations (lag 0–21 days) did not change when adding log-transformed population as an offset (S11 Fig).

## Discussion

To the best of our knowledge, this is the first study to investigate the temporal trends of the associations between temperature and hospitalisations for cardiovascular diseases and to explore the differences by demographic and geographic characteristics. For all groups, we found an increasing trend for the magnitude of associations between high temperature and hospitalisations for cardiovascular diseases and a decreasing trend for cold effects between 1995 and 2016. In subgroup analysis, the increasing magnitude of heat effects were larger (*p* = 0.002) in men than women and larger (*p* < 0.001) in people aged ≤69 years than in those aged ≥70 years. Our findings suggest a discernible sex-specific, age-specific adaptive pattern. Our results highlight the pressing need for preparations for emerging threats to cardiovascular health from hot temperatures.

The average effect of temperature on hospitalisations for cardiovascular diseases during 1995 and 2016 showed that cold temperatures were positively associated with hospitalisations for cardiovascular diseases. The cold effect was consistent with previous studies [24]. Phung and colleagues quantitatively analysed the association between temperature and hospitalisations for cardiovascular diseases, indicating the pooled risk of cardiovascular hospitalisations increased 2.8% for cold exposure (cold temperature defined as number of degrees below the defined threshold or average value or comparison between extreme cold condition and the reference value) [24]. Consistently, studies in the United States and in European cities also reported stronger cold effects than hot effects [25,26]. In our study, high temperature was not significantly associated with the increased risks of hospitalisations for cardiovascular diseases. The finding was in line with a range of previous research results, including meta-analysis [24,27] and original studies [14,15,28,29].

The associations between temperature and hospitalisations for cardiovascular diseases showed different patterns in 1995 and 2016 in our study. The results suggested a decreasing trend in the risks of hospitalisation for cardiovascular diseases associated with cold temperatures from 1995 to 2016, whereas the heat effect increased. A study conducted at the similar latitude, in the southeast of Brazil, also noticed a temporal increase in the association between heat exposure and hospitalisations during the 2000–2015 hot seasons [14]. Even though the 2 study areas have different demographic characteristics and different socioeconomic development status, we both observed a similar increasing trend of heat-related hospitalisations. However, another study in Spain compared temperature–cardiovascular hospitalisation associations in 1997–2002 and in 2004–2013. The study found a nonsignificant increase in cold-related hospitalisations for cardiovascular diseases and a nonsignificant reduction in heat effect in later period. The temporal variation in these 2 periods showed an opposite trend from our results [15]. That is probably due to different features of climate, human behaviour, and climate change adaptation policies in the 2 study areas. In addition, heat effect on mortality increased between 1993 and 2006 in Australia [21]. Under the present climate-changing scenario, Australia would be exposed to more extreme heat in the future [30], which will pose more burden to the healthcare system, especially the circulation-related healthcare facilities during hot days.

We found robust results amongst subgroups. A similar decreasing trend for cold effect and an increasing trend for heat effect was demonstrated in different sex, age, and climate zone groups between 1995 and 2016. For the heat effect, the increasing magnitude of RRs in people aged ≤69 years was higher (*p* < 0.001) than those aged ≥70 years, indicating the ≤69 years age group became more vulnerable to high temperatures. Previous studies have reported that the elderly are a susceptible population [31]. However, heat–mortality associations decreased in the elderly between 1987 and 2000 in 107 US cities [8], and the decreasing trend of the association was largest in the elderly in comparison with other age groups in the US and 15 Asian cities [5,10]. RRs of heat–mortality association decreased 48% for those over 65 years, whilst those for the 15–44 years age group only decreased 26% in New York City between the 1900s and 2000s [32]. Our results and other evidence agree that the elderly have adapted to high temperatures better than young adults.

The increasing magnitude of heat–hospitalisation associations for cardiovascular diseases was greater (*p* = 0.002) in men than that in women, indicating men were getting more susceptible to high temperatures than women in the 21-year study period. Previous studies have found that men across all ages showed increased risks of heat illness than women [33]. An animal mechanism study demonstrated the hyperthermic response of heat exposure is mediated by testosterone [34]. Physiological and behavioural factors could explain the increased heat association in men. The increasing magnitude of heat effects amongst different climate zones did not show much difference.

In the context of global warming, the people of Queensland benefit from a warmer winter, manifested by the decrease in cold–hospitalisation associations. However, the heat effect became more significant, even with increased access to air conditioning and improved living conditions, especially in men and people ≤69 years. This finding highlights the deleterious health effects of heat exposure. Reducing outdoor activity and outdoor work during high-temperature days could possibly protect these vulnerable population from hospital admissions for cardiovascular diseases.

There are strengths of this study. First, this study used 21 years of postal-area–level meteorological data and postcode-level hospitalisation data, which allow us to analyse precisely to detect small effect sizes. Secondly, this study employed validated statistical methods. The time-stratified case-crossover design can minimise autocorrelation effects and provide sufficient adjustments for confounders. This study provides additional evidence and significant insights for climate change adaptation. It provides improved estimates of the temperature-related hospitalisations for cardiovascular diseases.

There are also some limitations. First, we did not control for air pollution because the data were unavailable. However, because meteorological conditions can influence the concentrations of air pollutants whilst air pollutants could not influence meteorological conditions, air pollutants should not be treated as a confounder of ambient temperature and health effects. Previous studies have also found that the health impacts of ambient temperature changed minimally after adjusting for particulate matter and ozone [35]. Secondly, we used 2 coding systems to define cardiovascular diseases. Changing from ICD-9 to ICD-10 might cause coding inaccuracies. However, previous studies demonstrated that there were no discontinuities for switching from ICD-9 to ICD-10 for major cardiovascular disease subtypes [36]. Being limited by data-recording method, we could not separate first-time hospitalisation from repeated hospitalisation for cardiovascular diseases. Unfortunately, we did not have access to data on influenza in Queensland, Australia at the postcode level from 1995 to 2016. We cannot analyse the confounding effects of influenza. This design and analysis cannot confirm causal associations.

### Conclusions

Although we found a generally consistent decreasing trend in the associations between cold temperature and hospitalisations for cardiovascular diseases, no adaptation to high temperature was found during 1995–2016 in Queensland, Australia. The associations between high temperature and hospitalisations for cardiovascular diseases were actually increasing, especially for men and people ≤69 years old. This suggests a potentially worrying increase in vulnerability to heat-related cardiovascular morbidity in the context of global warming.

## Supporting information

S1 Fig Distribution of different climate zones in Queensland, Australia. The base map was downloaded from ASGS, Australian Bureau of Statistics (https://www.abs.gov.au/AUSSTATS/abs@.nsf/DetailsPage/1270.0.55.003July%202016?OpenDocument). The base map was open access. ASGS, Australian Statistical Geography Standard.
(TIF)

S2 Fig Exposure–response relationships between temperature and hospitalisations for cardiovascular diseases every 5 years with 3 years overlapping. Note: Dotted lines from left to right represent the first, tenth, 90^th^, and 99^th^ percentiles of temperature.
(TIF)

S3 Fig Exposure–response relationships between temperature and hospitalisations for cardiovascular diseases changing temperature lag days from 22 to 28. (TIF)

S4 Fig Exposure–response relationships between temperature and hospitalisations for cardiovascular diseases by changing temperature lag degree of freedom from 3 to 5. (TIF)

S5 Fig Exposure–response relationships between temperature and hospitalisations for cardiovascular diseases by changing the degree of freedom of relative humidity from 2 to 5. (TIF)

S6 Fig The lag–response relationships between cold temperature and hospitalisations predicted for 1995 and 2016, with 95% CI. CI, confidence interval.
(TIF)

S7 Fig The lag–response relationships between high temperature and hospitalisations predicted for 1995 and 2016, with 95% CI. CI, confidence interval.
(TIF)

S8 Fig The average cumulative exposure–response relationships between daily maximum temperature and hospitalisations for cardiovascular diseases along lag 0–21 days between 1995 and 2016 (A) and the cumulative exposure–response relationships between daily maximum temperature and hospitalisations for cardiovascular diseases along lag 0–21 days in 1995 (blue) and 2016 (red) (B). Note: polygon area represents 95% CI. CI, confidence interval.
(TIF)

S9 Fig The average cumulative exposure–response relationships between daily minimum temperature and hospitalisations for cardiovascular diseases along lag 0–21 days between 1995 and 2016 (A) and the cumulative exposure–response relationships between daily minimum temperature and hospitalisations for cardiovascular diseases along lag 0–21 days in 1995 (blue) and 2016 (red) (B). Note: polygon area represents 95% CI. CI, confidence interval.
(TIF)

S10 Fig The average cumulative exposure–response relationships between daily apparent temperature and hospitalisations for cardiovascular diseases along lag 0–21 days between 1995 and 2016 (A) and the cumulative exposure–response relationships between daily apparent temperature and hospitalisations for cardiovascular diseases along lag 0–21 days in 1995 (blue) and 2016 (red) (B). Note: polygon area represents 95% CI. CI, confidence interval.
(TIF)

S11 Fig The average cumulative exposure–response relationships between temperature and hospitalisations for cardiovascular diseases along lag 0–21 days between 1995 and 2016 after adding log-transformed population (A) and the cumulative exposure–response relationships between temperature and hospitalisations for cardiovascular diseases along lag 0–21 days in 1995 (blue) and 2016 (red) after adding log-transformed population (B). Note: polygon area represents 95% CI. CI, confidence interval.
(TIF)

S1 Table Comparison of the increasing magnitude of heat–hospitalisation associations for cardiovascular diseases amongst different subgroups. (XLSX)

S2 Table RRs (95% CIs) of the associations between cold temperatures (2.5^th^ and 5^th^ percentile of temperature against tenth percentile of temperature) and hospitalisations for cardiovascular diseases amongst different subgroups. CI, confidence interval; RR, relative risk.
(XLSX)

S3 Table RRs (95% CIs) of the associations between hot temperatures (95^th^ and 97.5^th^ percentiles of temperature against 90th percentile of temperature) and hospitalisations for cardiovascular diseases amongst different subgroups. CI, confidence interval; RR, relative risk.
(XLSX)

S1 Text STROBE statement. STROBE, Strengthening the Reporting of Observational Studies in Epidemiology.
(DOCX)

S2 Text Prospective analysis plan. (DOCX)

## Abbreviations

ASGS: Australian Statistical Geography Standard
CI: confidence interval
DLNM: distributed-lag nonlinear model
ICD: International Classification of Diseases
MHT: minimum hospitalisation temperature
QHAPDC: Queensland Hospital Admitted Patient Data Collection
RR: relative risk
SILO: scientific information for landowners
STROBE: Strengthening the Reporting of Observational Studies in Epidemiology
