# Supplementary figures and images for: Temporal trends of the association between ambient temperature and hospitalisations for cardiovascular diseases in Queensland, Australia from 1995 to 2016: A time-stratified case-crossover study
Source: PLoS Med. 2020 Jul 21;17(7):e1003176. doi: 10.1371/journal.pmed.1003176 (PMC7373260; doi:10.1371/journal.pmed.1003176)

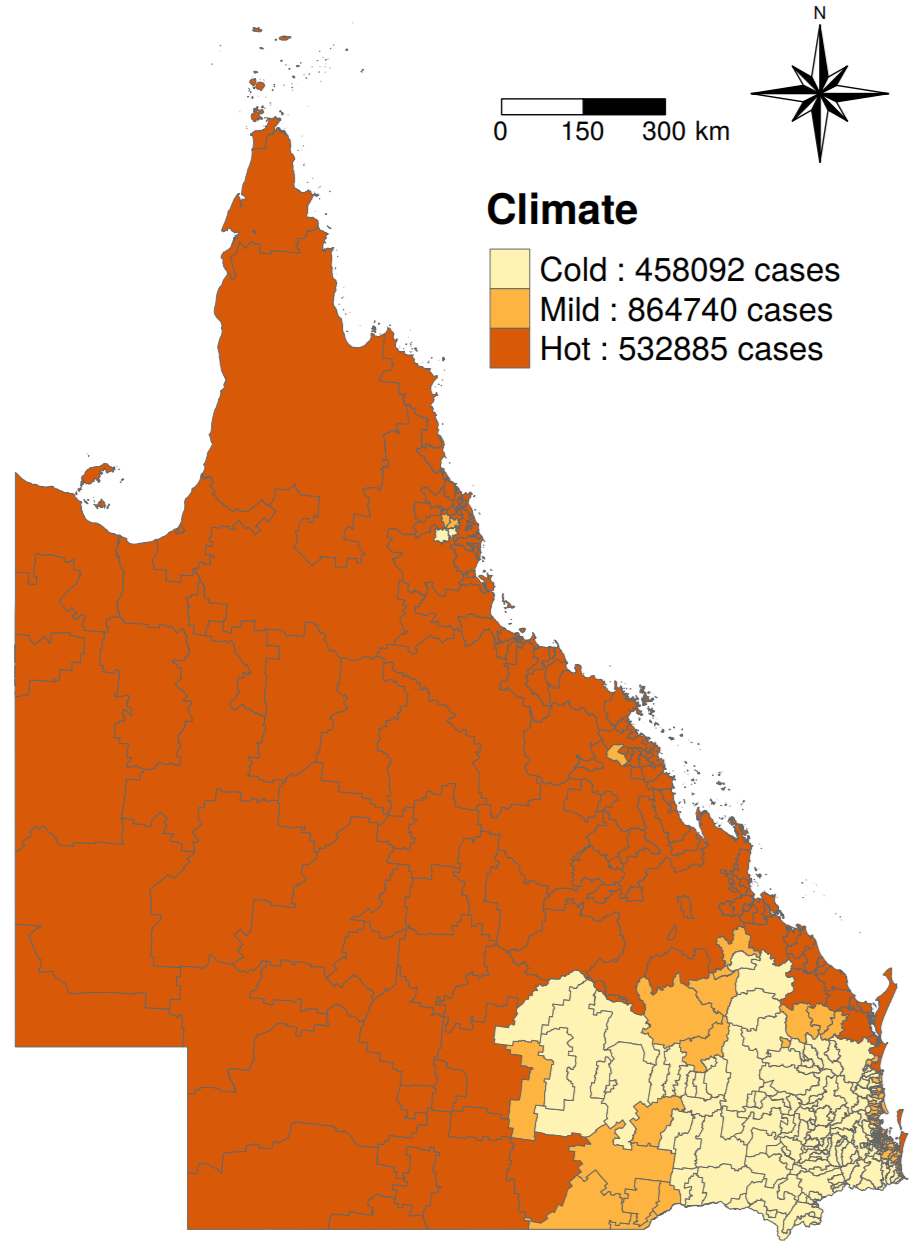

Supplement: S1 Fig — The base map was downloaded from ASGS, Australian Bureau of Statistics (https://www.abs.gov.au/AUSSTATS/abs@.nsf/DetailsPage/1270.0.55.003July%202016?OpenDocument). The base map was open access. ASGS, Australian Statistical Geography Standard. (TIF) [file pmed.1003176.s001.tif]

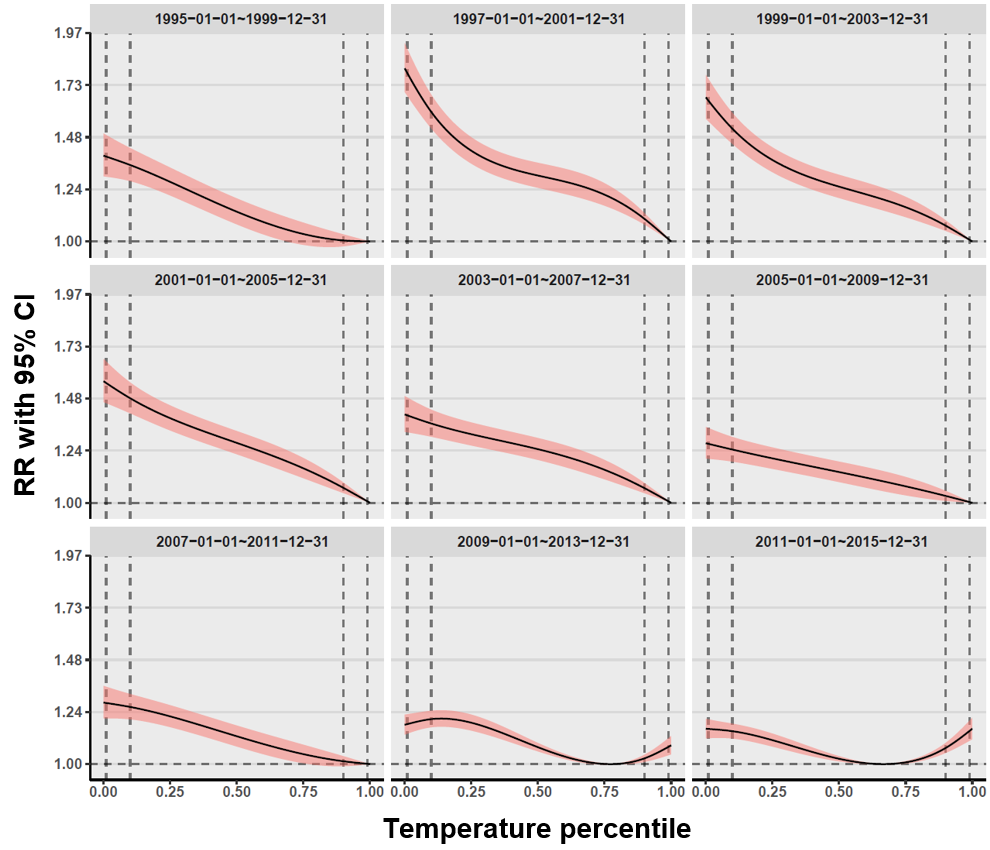

Supplement: S2 Fig — Note: Dotted lines from left to right represent the first, tenth, 90th, and 99th percentiles of temperature. (TIF) [file pmed.1003176.s002.tif]

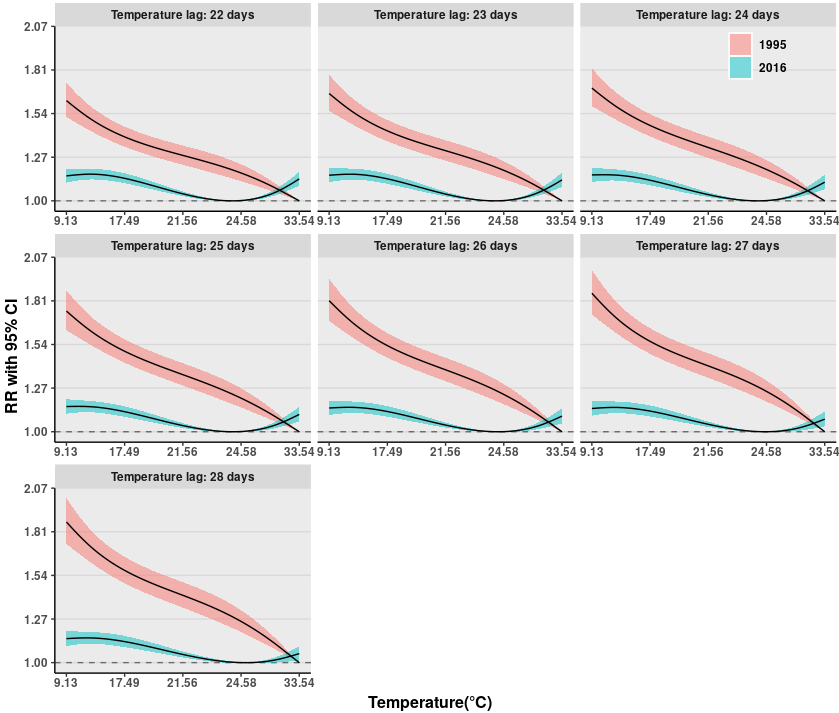

Supplement: S3 Fig — (TIF) [file pmed.1003176.s003.tif]

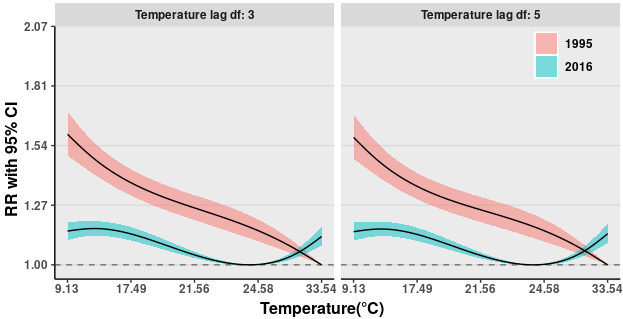

Supplement: S4 Fig — (TIF) [file pmed.1003176.s004.tif]

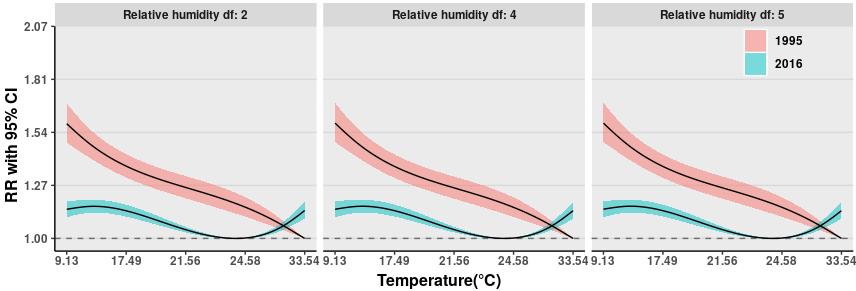

Supplement: S5 Fig — (TIF) [file pmed.1003176.s005.tif]

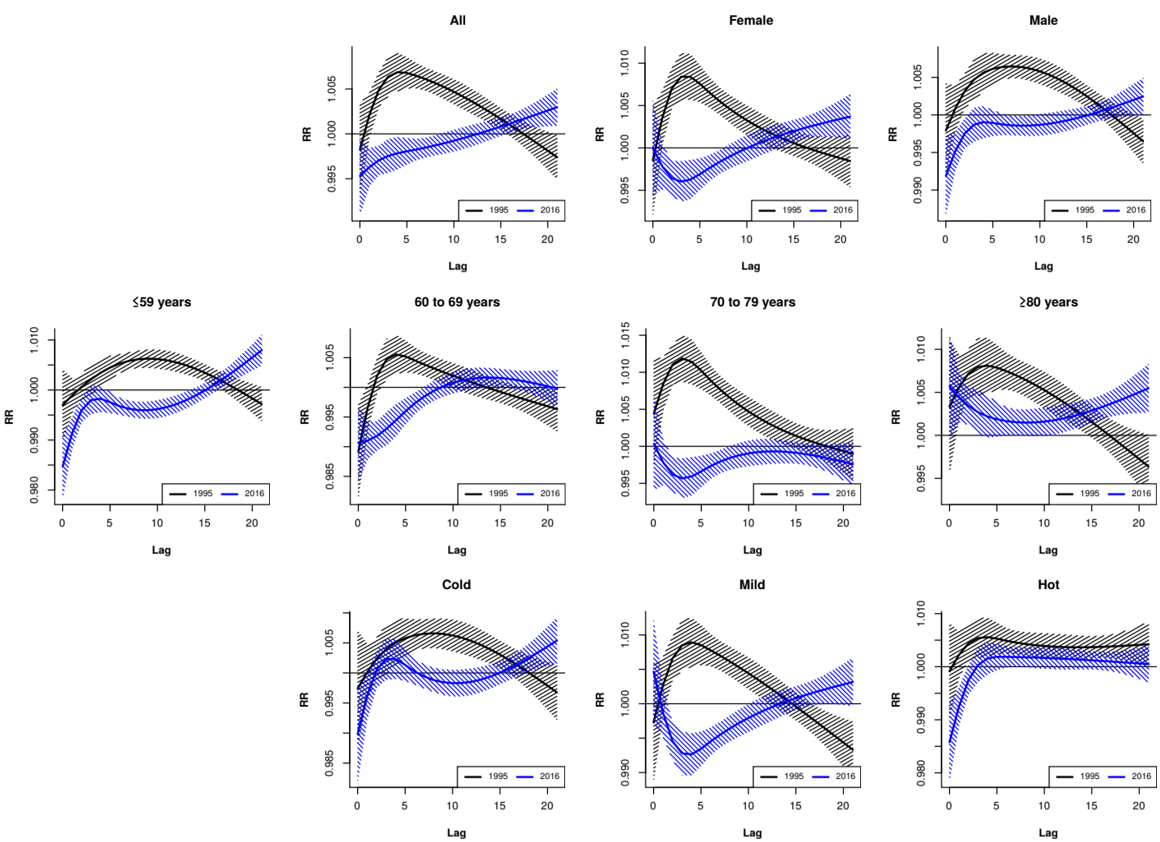

Supplement: S6 Fig — CI, confidence interval. (TIF) [file pmed.1003176.s006.tif]

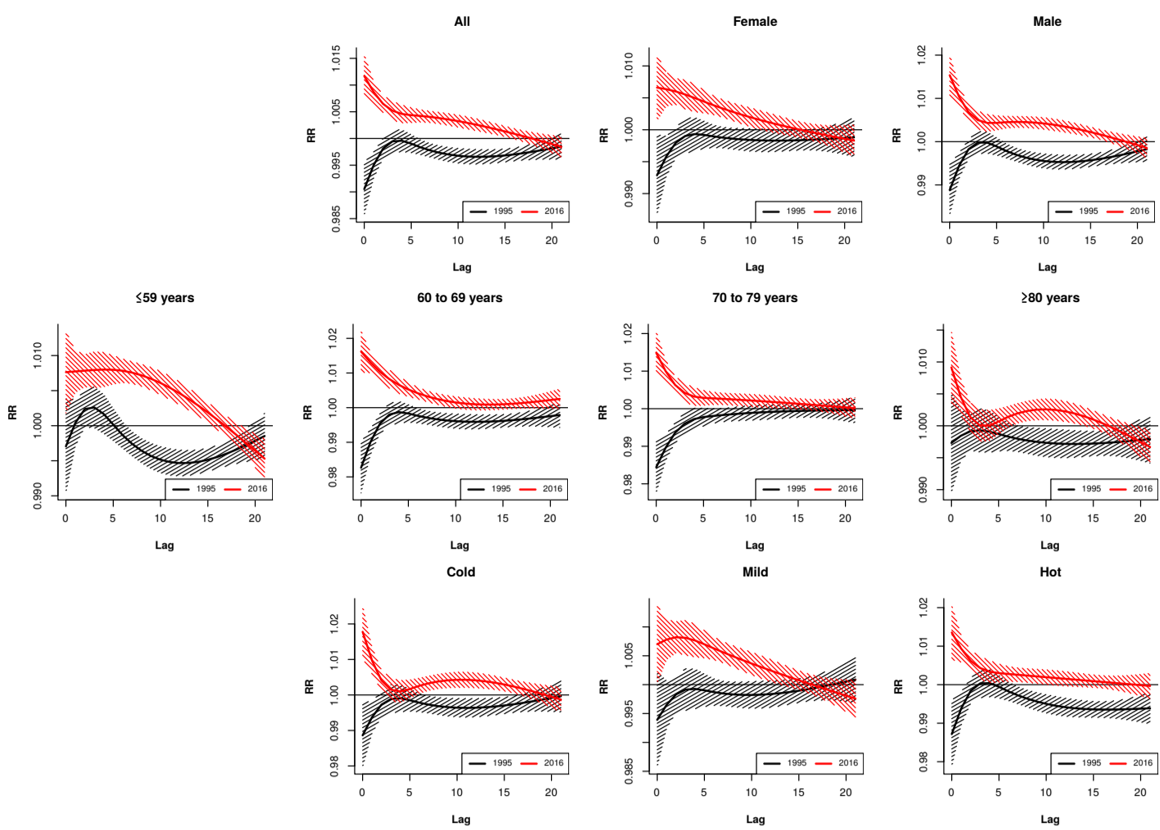

Supplement: S7 Fig — CI, confidence interval. (TIF) [file pmed.1003176.s007.tif]

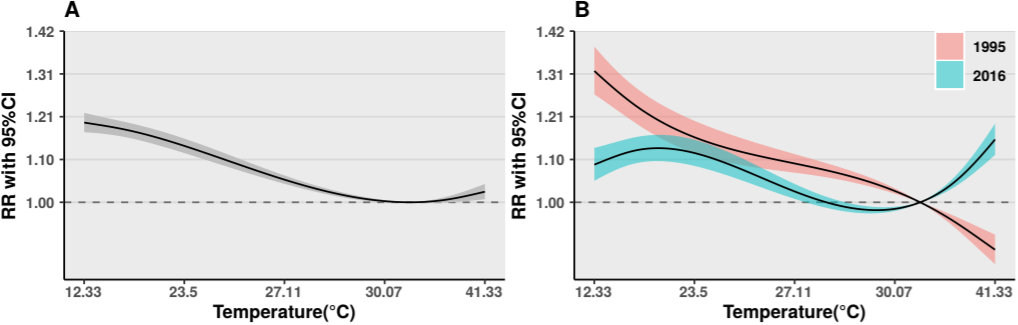

Supplement: S8 Fig — Note: polygon area represents 95% CI. CI, confidence interval. (TIF) [file pmed.1003176.s008.tif]

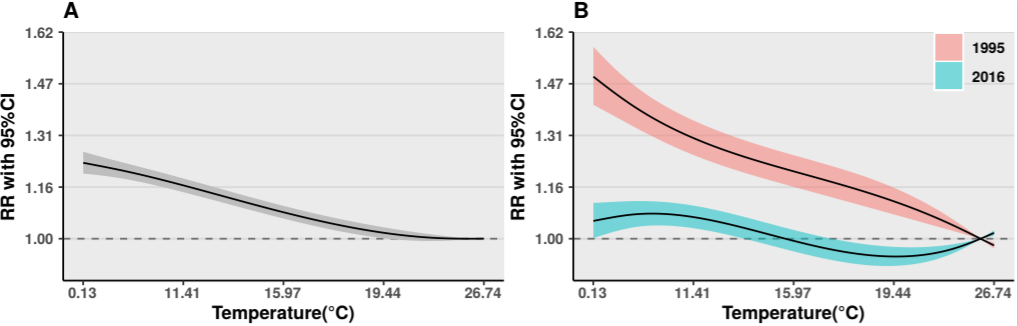

Supplement: S9 Fig — Note: polygon area represents 95% CI. CI, confidence interval. (TIF) [file pmed.1003176.s009.tif]

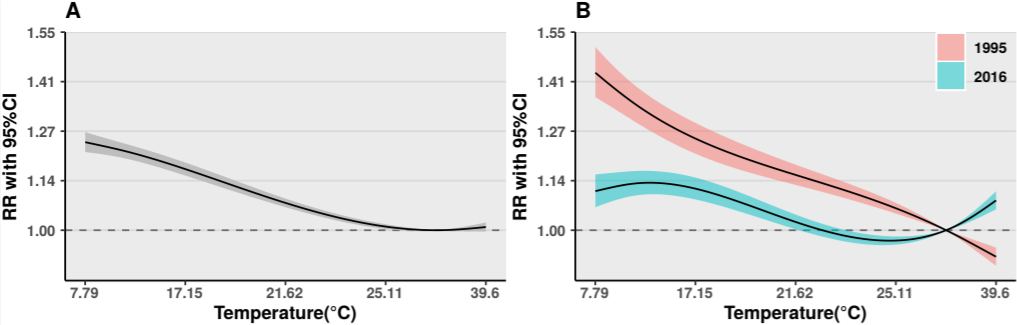

Supplement: S10 Fig — Note: polygon area represents 95% CI. CI, confidence interval. (TIF) [file pmed.1003176.s010.tif]

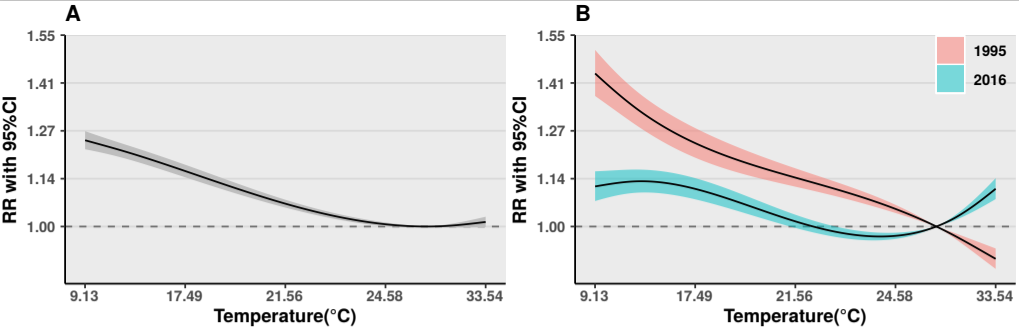

Supplement: S11 Fig — Note: polygon area represents 95% CI. CI, confidence interval. (TIF) [file pmed.1003176.s011.tif]
