# Supplementary material for: Temporal trends of the association between ambient temperature and hospitalisations for cardiovascular diseases in Queensland, Australia from 1995 to 2016: A time-stratified case-crossover study
Source: PLoS Med. 2020 Jul 21;17(7):e1003176. doi: 10.1371/journal.pmed.1003176 (PMC7373260; doi:10.1371/journal.pmed.1003176)
Supplement: S2 Text — (DOCX) [file pmed.1003176.s016.docx]

**S2 Text. Prospective analysis plan and modifications following comments from editors and reviewers**

**Date of commencing this research:** May 20, 2019.

**Research type:** A time-stratified case-crossover study

**Research objectives:**

1. To quantify the associations between ambient temperature and hospitalizations for cardiovascular diseases in Queensland, Australia between 1995 and 2016.

2. To investigate the temporal variations in the association between ambient temperature and hospitalizations for cardiovascular diseases in Queensland, Australia from 1995 to 2016

3. To explore whether the temperature-hospitalization associations varied across population subgroups (i.e. sex and age) and climate zones.

**Subjects:**

Postcode level hospitalization data of Queensland were collected between 1 January 1995 and 31 December 2016 from Queensland Hospital Admitted Patient Da ta Collection (QHAPDC), Queensland Health. The QHAPDC collects demographic data and clinical information on all patients admitted to public and licensed private hospitals and private day surgeries in Queensland, Australia.

**Exposure measurements:**

The maximum and minimum temperature datasets are 0.05° × 0.05° (about 5km×5km) grid data constructed from observational weather data from the Australian Bureau of Meteorology. Daily mean temperature, approximated as the mean of daily maximum and minimum temperatures were used to analyse the temperature-hospitalization associations

**Outcomes:**

We used the relative risks(RR) at 1st percentile of temperature against 10th percentile of temperature as cold effects, the RR at 99th percentile of temperature against 90th percentile of temperature as heat effects.

**Analyses:**

1. Descriptive analyses

Basic characteristics of hospitalizations for cardiovascular diseases and ambient temepratures in Queensland, Australia during the study period will be summarized, such as the number of hospitalizations, the sex and age distributions of hospitalizations, and the variation of the minimum, 25^th^ 50^th^ 75^th^ and maximum temperatures during the study period.

2. Statistical analyses

1) Temperature-hospitalization associations

We used a time-stratified case-crossover design to control for the long-term trend and seasonal trend. We performed conditional quasi-Poisson regression with distributed lag non-linear model (DLNM) to estimate the temperature-hospitalization associations, which were reported as relative risks (RRs). We also controlled for confounding factors (i.e., day of the week and public holidays).

2)Temporal trends in the temperature-hospitalization association

We examined the temporal variation of associations between ambient temperature and hospitalizations for cardiovascular diseases with time-varying DLNM. We extended the above time-constant model to the time-varying DLNM by including a linear interaction between time (a sequence from the first day to the last day of study period) and $cb(temp)$. We compared the RRs in the middle of year 1995 and the middle of year 2016, to examine whether there was a difference between the two years. Stratified analyses will be performed by sex (men and women), age groups (0-59, 60-69, 70-79, ≥80 years) and climate zones.

3. Additional analyses

Sensitivity analyses will be performed to test the robustness of our findings.

**Modification based on the comments from editors and reviewers:**

Following the suggestions of reviewers, we presented detailed description for the definition of minimal hospitalization temperature (MHT) and we did sensitivity test by using different cut-offs (2.5^th^ 5^th^ 97.5^th^ and 95^th^ percentile) of temperature and alternative daily temperature metrics (e.g. maximum, minimum, apparent temperature).
